# Progress in Increasing Electronic Reporting of Laboratory Results to Public Health Agencies — United States, 2013

**Published:** 2013-09-27

**Authors:** Kathryn Turner, Janet Hamilton, C. Jason Hall, Robert W. Pinner, Kathy Gallagher, Laura Conn

**Affiliations:** Idaho Div of Public Health; Florida Dept of Health; Div of Preparedness and Emerging Infections; National Center for Emerging and Zoonotic Infectious Diseases; Div of Health Informatics and Surveillance (proposed), Center for Surveillance, Epidemiology and Laboratory Svcs (proposed); Office of Public Health Scientific Svcs (proposed), CDC

Electronic reporting of laboratory results to public health agencies can improve public health surveillance for reportable diseases and conditions by making reporting more timely and complete (1). Since 2010, CDC has provided funding to 57 state, local, and territorial health departments through the Epidemiology and Laboratory Capacity for Infectious Diseases cooperative agreement to assist with improving electronic laboratory reporting (ELR)* from clinical and public health laboratories to public health agencies. As part of this agreement, CDC and state and large local health departments are collaborating to monitor ELR implementation in the United States by developing data from each jurisdiction regarding total reporting laboratories, laboratories sending ELR by disease category and message format, and the number of ELR laboratory reports compared with the total number of laboratory reports. At the end of July 2013, 54 of the 57 jurisdictions were receiving at least some laboratory reports through ELR, and approximately 62% of 20 million laboratory reports were being received electronically, compared with 54% in 2012. Continued progress will require collaboration between clinical laboratories, laboratory information management system (LIMS) vendors, and public health agencies.

Monitoring of ELR progress began in 2012 with creation of a list of laboratories for each jurisdiction based on 2010 data from the Clinical Laboratory Improvement Amendments database of certified laboratories and the American Hospital Association directory of laboratory facilities. To date, these lists, which have been further refined by public health agencies, identify approximately 10,400 laboratories that send reportable results to public health agencies nationwide. Of these, approximately 5,320 (51%) are hospital laboratories, 420 (4%) are facilities owned by one of four large commercial laboratories,† 400 (4%) are public health laboratories, and 4,260 (41%) are other laboratories, including small or regional commercial, specialty, and federal (including CDC and the Veterans Administration) laboratories. Of the 10,400 reporting laboratories, approximately 5,400 (52%) are considered priority targets§ for ELR by health departments. Through quarterly telephone calls and e-mails, CDC and public health agency staff members compile information about laboratory results reporting, including an annual estimate of the volume of reports.

As of July 31, 2013, a total of 54 of the 57 jurisdictions (48 state and six large local health departments) were receiving at least some laboratory reports through ELR. Almost 2,900 (28%) laboratories (52% of targeted laboratories) reported to at least one public health agency through ELR.¶ Based on 12-month estimates provided by 54 jurisdictions, approximately 62% of total laboratory reports are being received electronically. The proportion of laboratory reports received electronically varied by jurisdiction; 14 jurisdictions received >75% of laboratory reports electronically, and nine received <25% of reports electronically (Figure). Of all reports received electronically, 40% come from one of the four large commercial laboratories, 14% from the approximately 5,300 hospital laboratories, and 30% from public health laboratories. The proportion of reports received electronically also varied by disease category. For example, approximately 76% of reportable laboratory results for general communicable diseases were received through ELR. In contrast, a lower proportion of human immunodeficiency virus (HIV) and sexually transmitted disease (STD) reports (54% and 63%, respectively) were sent electronically, even though overall reporting volumes for these conditions were higher.

## Editorial Note

State and local public health departments have made substantial progress in ELR in recent years; 54 state and local public health departments now receive laboratory reports electronically, compared with 26 in 2005 (2). In the last year alone, the percentage of laboratory reports received electronically has increased 8 percentage points, from approximately 54% to 62%, and three states have begun receiving their first ELR transmissions.

The inclusion of electronic reportable laboratory results in the Centers for Medicare & Medicaid Services Electronic Health Record Incentive Program’s “meaningful use” requirements is advancing ELR implementation by providing incentives to hospitals that receive Medicare and Medicaid reimbursements and creating additional funding sources for activities related to ELR implementation. CDC has provided support to public health agencies and hospital laboratories for establishing meaningful use–compliant ELR transmissions through the Health Information Technology for Economic and Clinical Health component of the American Recovery and Reinvestment Act (3). This support includes outreach provided to hospitals, particularly critical access and rural hospitals, by the Laboratory Interoperability Cooperative (a consortium of Surescripts, the College of American Pathologists, and the American Hospital Association). A doubling in the number of hospitals sending finalized ELR transmissions using meaningful use standards during March 2012–July 2013 (Division of Preparedness and Emerging Infections, National Center for Emerging and Zoonotic Infectious Diseases, CDC, unpublished data, 2013) suggests that meaningful use might already be having an impact on ELR implementation. ELR implementation by hospitals is likely to accelerate as meaningful use moves into its next stage, in October 2013, when ELR changes from “menu,” or optional, to “core,” or required, for eligible hospitals to receive their incentives.

Various other efforts are contributing to implementation of ELR in the United States. During 2010–2012, a CDC and Council of State and Territorial Epidemiologists ELR task force developed products and tools (4) to help inform ELR implementation, including a table for associating reportable conditions with standard codes for test names and results (i.e., reportable conditions mapping tables) (5), a process checklist for ELR implementation (6), a report of legal considerations for states implementing ELR (7), and white papers on working with large laboratories (8) and LIMS vendors (9) to improve ELR. At CDC, enhanced communication and collaboration among CDC programs that provide funds to public health departments are helping to reinforce standards-based ELR implementation and ensure that ELR efforts are not duplicative. In addition, CDC is working with the Association of Public Health Laboratories to offer technical assistance to advance ELR through targeted, short-term implementation projects. Since January 2012, CDC has received 70 requests for ELR technical assistance from 30 jurisdictions; of 56 approved projects, 43 are either under way or completed. Examples include establishing ELR feeds to health departments from the four large laboratories, smaller regional laboratories, and public health laboratories and improving the processing and increasing the use of ELR for all conditions.

Substantial work remains, however, to achieve full and effective ELR implementation. Nearly three fourths of reporting laboratories, including half of those that are priority targets, still are not reporting electronically, so increasing the number of laboratories sending reports electronically is a key objective. In addition, effective ELR implementation will require that many public health agency disease surveillance information systems develop capacity to incorporate electronic reports efficiently. This is especially true for those systems used for conditions with high laboratory report volume, such as HIV and STDs. Moreover, public health agencies, laboratories, and LIMS vendors should work together to achieve consistent and accurate use of standardized vocabulary, to ensure that all reports are sent and that they are complete, and to reduce inessential state-to-state variability in electronic disease reporting requirements.

Longer term, public health agencies, clinical laboratories, and CDC should collaborate to devise strategies to stimulate and facilitate more rapid, complete, and effective ELR implementation. Such strategies could include improving coordination of ELR delivery from the large laboratories to public health agencies (e.g., exploring the use of single, multijurisdiction transmissions through a shared services environment), incorporating ELR capability in the products of LIMS vendors, developing information exchange with electronic health records, and capitalizing on the development of health information exchanges where possible.

What is already known on this topic?

Electronic reporting of laboratory results to public health agencies can improve public health surveillance for reportable diseases and conditions.

What is added by this report?

As of July 2013, a total of 54 state and large local public health agencies in the United States were receiving reports electronically for infectious diseases, compared with 26 in 2005. Approximately 62% of total laboratory reports in the United States were being sent electronically.

What are the implications for public health practice?

Progress in electonic laboratory reporting has resulted from a new emphasis and improved capacity and preparedness in health departments to address technical and policy issues. Continued progress will require collaboration between clinical laboratories, laboratory information management system vendors, and public health agencies, including improving the ability of disease surveillance information systems to effectively manage electronic reports.

## Figures and Tables

**FIGURE:**
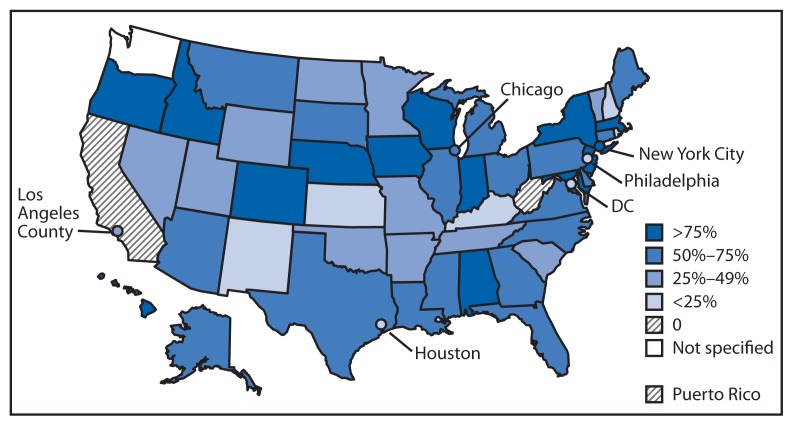
Percentage of laboratory reports received by public health agencies through electronic laboratory reporting — United States, 2013* * N = 57 jurisdictions, including 50 states, one territory, and six cities (for this report, Los Angeles County and the District of Columbia are categorized as cities). Data for Los Angeles County, which has a separate health jurisdiction, are not included in the data for California, which is expecting its first electronic laboratory report in October.

## Footnotes

* ELR generally refers to the automated messaging of laboratory reports, using HL7 or other formats and one or more electronic communication protocols. Direct web entry (i.e., manual entering of reports over the Internet by laboratories but not through electronic messaging) is included in this report as ELR because it does not require manual data entry by public health agencies into a disease surveillance information system or into an ELR repository.

† LabCorp, Quest Diagnostics, ARUP Laboratories, and Mayo Clinic.

§ Generally defined by jurisdictions as laboratories that send enough reportable results to a jurisdiction to make establishing automated transmission of an ELR file worthwhile.

¶ In 22 jurisdictions, 1,038 laboratories reported using direct web entry for at least some reports.
